# Grifolic acid induces GH3 adenoma cell death by inhibiting ATP production through a GPR120-independent mechanism

**DOI:** 10.1186/s40360-018-0215-4

**Published:** 2018-05-30

**Authors:** Yufeng Zhao, Lei Zhang, Aili Yan, Di Chen, Rong Xie, Yingguang Liu, Xiangyan Liang, Yanyan Zhao, Lanlan Wei, Jun Yu, Xi Xu, Xingli Su

**Affiliations:** 1 The institute of Basic Medical Sciences, Xi’an Medical University, Xi’an, 710021 China; 2 Department of Gerontological Surgery, The First Affiliated Hospital, Xi’an Medical University, Xi’an, 710061 China; 3 Medical Research Center, The Second Affiliated Hospital, Xi’an Medical University, Xi’an, 710038 China

**Keywords:** Grifolic acid, GH3 cells, Mitochondria, Cell death

## Abstract

**Background:**

Grifolic acid is a derivative of grifolin, an antitumor natural compound, and it was reported as an agonist of free fatty acid receptor GPR120. Little is known about its antitumor effects and the involvement of GPR120.

**Methods:**

GH3 cells, the rat anterior pituitary adenoma cells, were cultured and the cell death was measured by MTT assay and Annexin V/PI staining. The mitochondrial membrane potential (MMP) of GH3 cells was measured by JC-1 staining. Cellular ATP levels and the intracellular NAD/NADH ratio were measured. GPR120 expression in GH3 cells was observed by RT-PCR and Western Blot, and siRNA was used to inhibit GPR120 expression in GH3 cells.

**Results:**

Grifolic acid dose- and time-dependently induced the necrosis of GH3 cells. Grifolic acid significantly reduced the mitochondrial membrane potential (MMP) and decreased cellular ATP levels in GH3 cells. In contrast, the MMP of isolated mitochondria was not decreased by grifolic acid. The intracellular NAD/NADH ratio was significantly increased by grifolic acid. GPR120 is expressed in GH3 cells, but GPR120 agonists such as EPA, GW9508 and TUG891 did not affect the viability of GH3 cells. Moreover, GPR120 siRNA knockdown showed no significant influence on grifolic acid-induced GH3 cell death.

**Conclusion:**

Grifolic acid induces GH3 cell death by decreasing MMP and inhibiting ATP production, which may be due to the inhibition of NADH production through a GPR120-independent mechanism.

**Electronic supplementary material:**

The online version of this article (10.1186/s40360-018-0215-4) contains supplementary material, which is available to authorized users.

## Background

Some natural compounds, which are isolated from botany, fungi and marine organism, are putative candidates for antitumor drugs [1]. It is well known that grifolin, one of the natural compounds isolated from the fresh fruiting bodies of the mushroom *Albatrellus confluens* [2], exhibits antitumor effects on nasopharyngeal carcinoma, osteosarcoma, and gastric tumor cells [3–5]. Grifolic acid (the structure in Additional file 1) is a derivative of grifolin, but its effects on tumor cells are not well established. Some reports showed that grifolic acid was an agonist of free fatty acid receptor GPR120 [6], and grifolic acid was able to activate extracellular regulated protein kinases (ERK1/2), causing increased secretion of glucose-dependent insulinotropic polypeptide (GIP) from GPR120-expressing enteroendocrine cells [7]. It was also showed that GPR120 activation might produce protective effects on murine enteroendocrine cell line STC-1 cells [8]. The effects of grifolic acid on tumor cells and the involvement of GPR120 warrants further study.

Anterior pituitary adenomas, one of the common intracranial tumors, is increasingly diagnosed due to the advances in neuroimaging technology [9]. Besides trans-sphenoidal surgery, medical therapies are important treatments for anterior pituitary adenomas [10]. New effective antitumor drugs may significantly improve the therapy of anterior pituitary adenomas. In this study, we observed the effects of grifolic acid on the viability of GH3 cells, the rat anterior pituitary adenoma cells that secret growth hormone and prolactin [11].

The death of tumor cells is often related to the dysfunction of mitochondria. Mitochondria are essential to produce ATP and play a dominant role in cellular viability, apoptosis and death [12]. Intracellular ATP at the normal level is required for cell survival, and the reduction of ATP level results in the apoptosis or necrosis of living cells [13, 14]. Mitochondrial membrane potential (MMP), which is generated during the procedure of redox energy transfer from NADH to oxygen via the electron transport chain in mitochondria, represents the function of mitochondria and is critical for ATP production. The actions of grifolic acid on mitochondria function such as MMP and ATP production were also investigated in this study. In addition, we found GPR120 expression in GH3 cells, and the role of GPR120 in the effects of grifolic acid on GH3 cells was studied.

## Methods

### Chemicals

Grifolic acid and TUG891 were obtained from R&D Inc. (Minnneapolis, USA). GW9508, EPA, GPR120 polyclonal antibody, MTT and Cellular ATP assay kits were bought from Sigma-Aldrich (St. Louis, USA). Annexin V-FITC/PI staining kits were the products of BD Pharmingen (San Jose, USA). Rat GPR120 siRNA, lipofectamine RANiMAX, DMEM, FBS, JC-1 and Mitochondria Isolation Kit for Culture Cells were obtained from Thermo Fisher Scientific (Waltham, USA). NAD/NADH Assay Kits were the products of Abcam (Cambridge, UK). Protein extraction kits were bought from Bio-Rad (Hercules, USA). RNA isolation kits, reverse transcription kits and PCR kits were the products of Takara Biotechnology (Dalian, China).

### Cell culture

GH3 cells were obtained from American Type Culture Collection (ATCC Number: CCL-82.1™) and cultured in DMEM containing 10% FBS, 100 U/ml penicillin and 100 μg/ml streptomycin. The media were changed every 2 days, and GH3 cells were sub-cultured after 80% confluence and seeded to plates or dishes for the following measurements.

### Cell viability assay

GH3 cells grew up to 90% confluence in 96-well plates and then were changed to serum-free medium with regent treatment including grifolic acid, EPA, GW9508 and TUG891. At the end of treatment, MTT was added into media at a final concentration of 0.5 mg/ml. Four hours later, the media were discarded and 100 μl isopropanol with 0.01 mol/L HCl was added to each well. After the formazan crystals were fully solubilized, the absorbance values at 560 nm were measured by ELISA reader (Thermo Fisher, USA). The background absorbance values at 630 nm were also measured and subtracted from that of 560 nm. Then the absorbance values were used for statistical analysis. The experiments were performed in triplicate.

### Flow cytometry analysis of cell death

After being treated by grifolic acid in serum-free medium, GH3 cells were detached from the dishes by 0.05% trypsin/EDTA and stained using AnnexinV-FITC/PI staining kits [15]. Briefly, the cells were re-suspended into the binding buffer at 1×10^6^ cells/ml, and AnnexinV-FITC/PI was added to cell suspension in a dilution of 1:20. The cells were gently mixed and incubated for 15 min at room temperature in the dark. Finally, the cells were diluted into binding buffer and went through the flow cytometry to measure AnnexinV- and PI-staining positive cells (BD Biosciences, USA). The experiments were performed in triplicate.

### Cellular ATP measurement

Cellular ATP levels in GH3 cells were measured using ATP detection assay kits [16]. Briefly, GH3 cells after being treated by grifolic acid in serum-free medium were lyzed by detergent under shaking at 700 rpm for 5 min. The constituted substrate solutions were added for incubation for 5 min in a dark place. Then the luminescence of each sample was recorded using the luminescence plate reader (Thermo Fisher, USA). The standard curves were constructed and the ATP level of each sample was calculated. The total protein levels were quantified by BCA assay and used to correct the cellular ATP levels for data analysis. The experiments were performed in triplicate.

### NAD/NADH measurement

GH3 cells (4×10^5^ cells per sample) were washed in cold PBS and treated with NAD/NADH Extraction Buffer. The buffer was centrifuged at 12,000 g for 5 min at 4 °C and the supernatants were collected for NAD/NADH assay [17]. As indicated by the instruction, each sample was divided into two parts for NADt (total NAD including NAD and NADH) assay and NADH assay respectively. The samples were mixed with Reaction Mix firstly and incubated for 5 min at room temperature. Then NADH Developer was added to incubate for 4 h. The OD value of each sample was read at 450 nm. NAD/NADH ratio was calculated as: NAD/NADH ratio = (NADt-NADH) / NADH. The experiments were performed in triplicate.

### MMP measurement

GH3 cells were stained with JC-1 (a final concentration of 5 μg/ml) in the serum-free medium for 15 min at 37 °C in a humidified 5% CO_2_ incubator. Then the cells were washed with the serum-free medium and put onto the stage of confocal microscope (Leica TCS SP8). The fluorescence intensity was recorded every 5 min before and after the treatment with grifolic acid. The red fluorescence (excitation wavelength at 560 nm and emission wavelength at 600 nm) and green fluorescence (excitation wavelength at 488 nm and emission wavelength at 535 nm) were measured synchronously, and the ratio of fluorescent intensity (red/green) for each cell was analyzed using the LAS LITE software [18]. The experiments were performed in triplicate.

### Mitochondria isolation

The mitochondria were extracted from GH3 cells using the Mitochondria Isolation Kit for the Culture Cells under the instruction [19]. GH3 cells (1×10^7^ cells per sample) were loaded with JC-1 firstly and then were harvested to isolate mitochondria. Mitochondria isolation reagent A was added to incubate on ice for 2 min, followed by the adding of mitochondria isolation reagent B and C. Then the mixtures were centrifuged at 700 g for 10 min at 4 °C. The supernatants were collected and centrifuged at 12,000 g for 15 min at 4 °C, and the pellets were collected and incubated in mitochondria isolation reagent C for MMP measurement.

### GPR120 siRNA transfection

GH3 cells grew to 70% confluent at the time of transfection. The transfection complexes for rat GPR120 Silencer Select siRNA (siRNA ID: S148233, Invitrogen) and Lipofectamine RNAiMAX were prepared by mixture of siRNA (100 pmol in 50 μl of Opti-MEM medium) and Lipofectamine RNAiMAX (1 μl in 50 μl of Opti-MEM medium). The complexes were added to the medium at a dilution of 1:4. The cells were cultured for 48 h and then GPR120 knockdown was measured by Western blot. The cells with transfection were used to measure cell death, cellular ATP levels and MMP.

### Western blot

In brief, GH3 cells were homogenized and total protein was extracted using ReadyPrep protein extraction kits and quantified by BCA assay. The protein (40 μg) was analyzed by SDS-PAGE on a 10% Peptide Criterion Gel and transferred to nitrocellulose membranes using a Trans-Blot SD semi-dry electrophoresis transfer cell (Bio-Rad). The membranes were then probed with rabbit-anti GPR120 antibody (1:1000) and the blot was developed with the chemiluminescent reagents. The luminescence of the membranes was imaged by the ChemiDoc MP gel imaging analysis system. The experiments were performed in triplicate.

### RT-PCR

The total RNA from GH3 cells were extracted and reverse transcription was performed using the kits from Takara. cDNA was used to run PCR with rat GPR120 primers. The forward primer is 5′- GCA TAG GAG AAA TCT CAT GG-3′ and the reverse primer is 5′- GAG TTG GCA AAC GTG AAG GC-3′. The PCR product size was 340 bp. The reaction mixture was denatured for 5 min at 94 °C for 30 s, and then it went to 30 cycles of denaturing at 94 °C for 30 s, annealing at 58 °C for 15 s and extending at 72 °C for 15 s. PCR products were analyzed by electrophoresis on 1.4% agarose gel with ethidium bromide. The experiments were performed in triplicate.

### Data analysis

The data were expressed as Mean ± S.EM. D’Agostino-Pearson omnibus test was used to test the normality of data distribution. For the normal distribution data, comparisons of means of multiple groups with each other or against one control group were analyzed with one way ANOVA followed by the appropriate *post-hoc* tests. For the abnormal distribution data, Kruskal-Wallis H test was used to analyze the significance of groups. *P* < 0.05 was considered to be significantly different.

## Results

### Effects of grifolic acid on GH3 cell viability

**Fig. 1:**
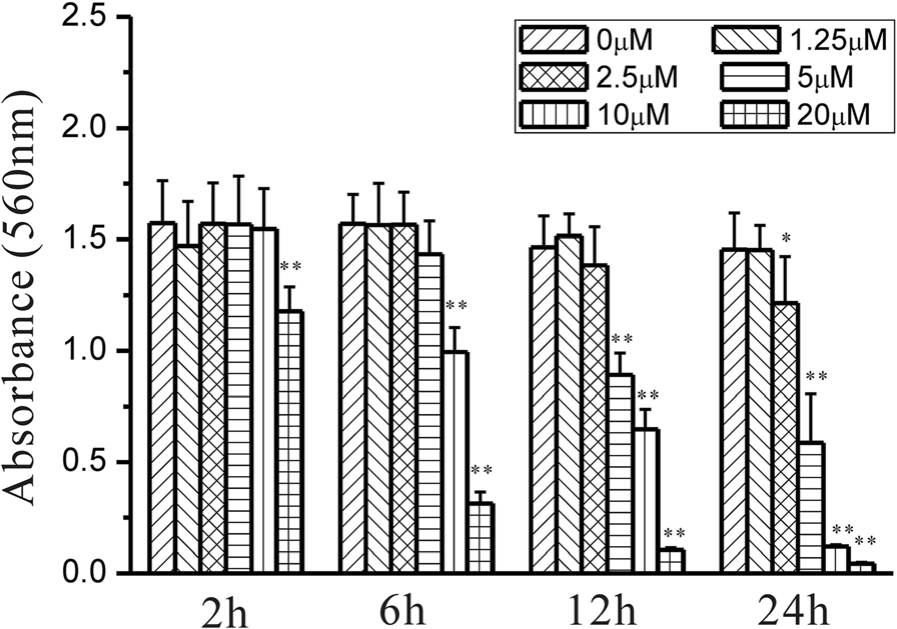
Grifolic acid reduces GH3 cells viability. GH3 cells in serum-free medium were treated by grifolic acid for different time, and then MTT assay was used to measure cell viability. The absorbance values of MTT assay at 560 nm were analyzed. * means *P* <0.05 vs control. ** means *P* < 0.01 vs control, *n* = 16

**Fig. 2:**
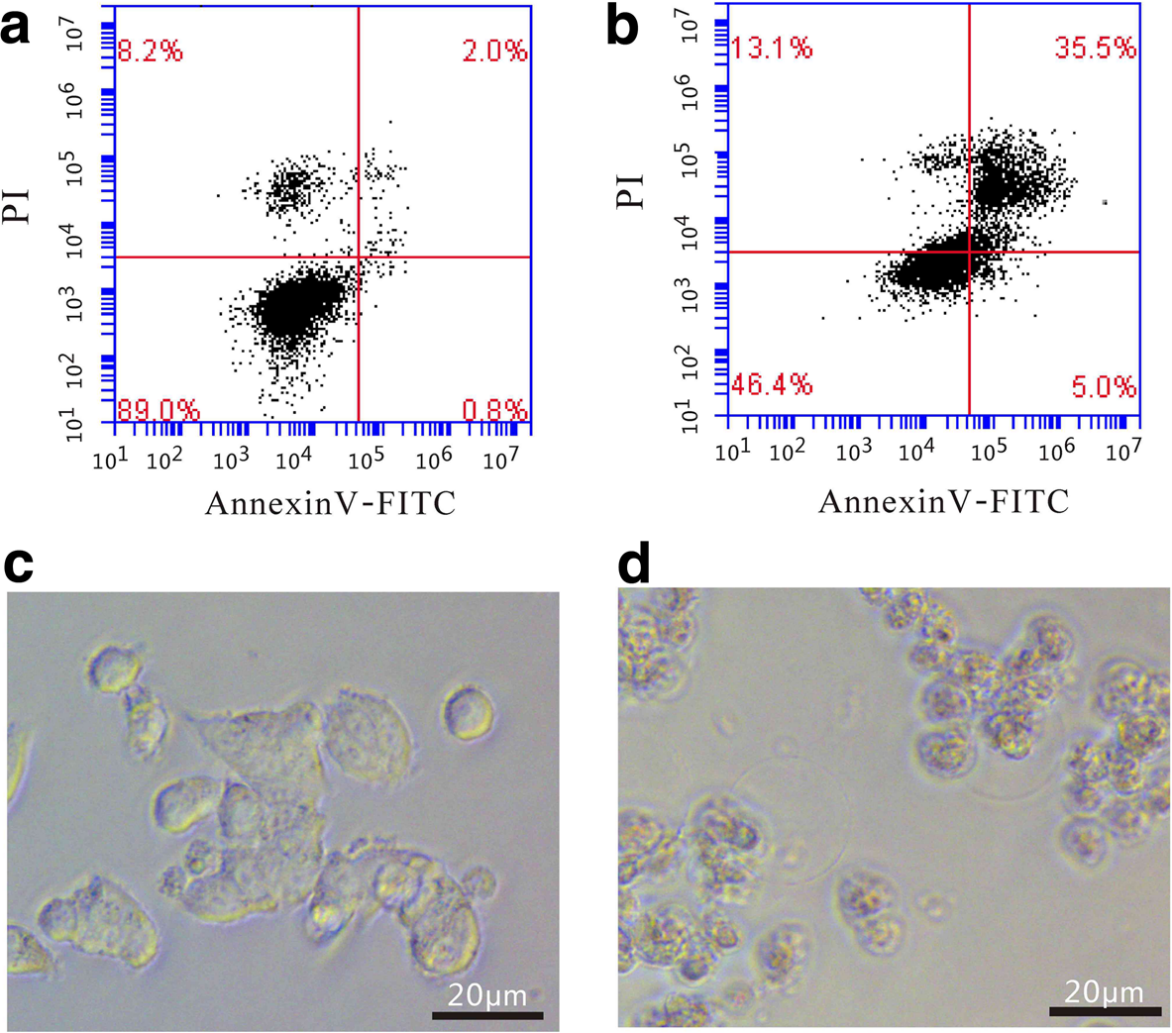
Grifolic acid induces cell death of GH3 cells. **a** Flow cytometry measurement showed that GH3 cells in control had low level of cell death as indicated by low staining of Annexin V and PI; **b** GH3 cell after grifolic acid treatment (10 μmol/L for 6 h) showed a significant increase in cell necrosis as indicated by high percentage of Annexin V and PI-positive staining cells; **c** The normal shape of GH3 cells in control; **d** GH3 cells showed swelling and broken after grifolic acid treatment. The photos were representative results of 3 independent experiments

Grifolic acid (20 μmol/L) inhibited the viability of GH3 cells after 1 h incubation and resulted in total cell death 6 h later. It showed a dose- and time-dependent inhibition of GH3 cell viability from 2.5 μmol/L to 20 μmol/L in 24 h incubation (Fig. 1). The IC50 of grifolic acid at 24 h treatment was 4.25 μmol/L. AnnexinV/PI staining and the flow cytometry analysis was also used to observe the death of GH3 cells. The percentage of Annexin V and PI-positive GH3 cells in control was 2.45% ± 0.83%, and it increased to 42.51% ± 7.86% after treatment with grifolic acid (10 μmol/L) for 6 h. The represent analysis results were shown in Fig. 2a and b. GH3 cells in control showed normal shape (Fig. 2c), but they were significantly swollen and mostly broken after grifolic acid treatment (Fig. 2d).

### Effects of grifolic acid on MMP and ATP production in GH3 cells

**Fig. 3:**
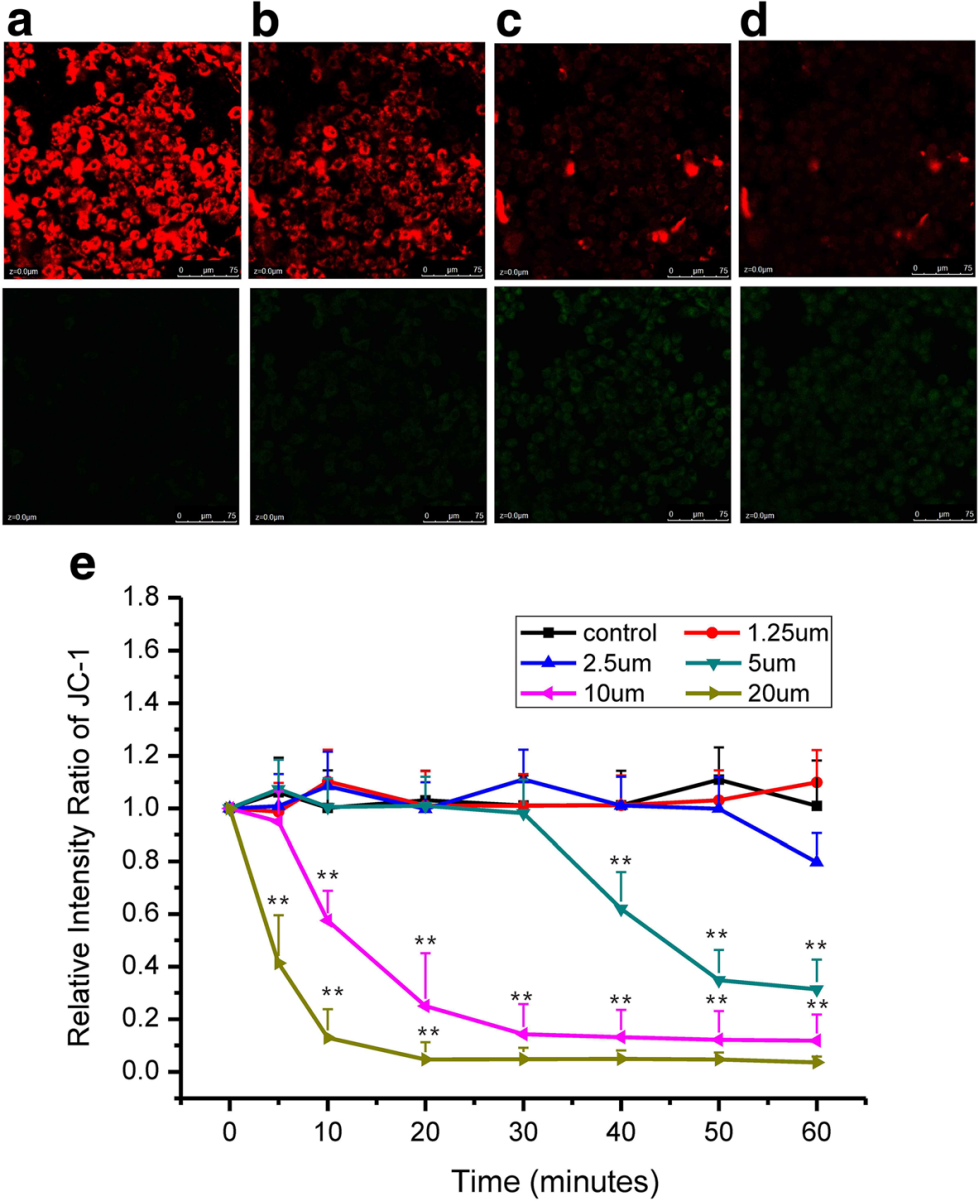
Grifolic acid diminishes MMP of GH3 cells. The fluorescent intensity of MMP indicator JC-1 in control (**a**) and after 20 μmol/L grifolic acid treatment for 5 min (**b**), 10 min (**c**) and 20 min (**d**). The statistical analysis of fluorescent intensity ratio of JC-1 in each cell was shown in (**e**) to reflect MMP levels. ** *P* < 0.01 vs control, *n* = 80

MMP was indicated by JC-1 fluorescence [20]. JC-1 exhibited membrane potential-dependent accumulation in mitochondria, showing the shift of emission wavelength from green to red. Mitochondrial depolarization was indicated by a decrease in the red/green fluorescence intensity ratio [21]. We found that grifolic acid resulted in a significant decrease in the red/green fluorescence intensity ratio in a dose-dependent and time-dependent manner. Grifolic acid (20 μmol/L) significantly attenuated MMP after 5 min incubation and caused the maximal effect in 20 min. Grifolic acid (10 μmol/L) also took effects in 10 min and achieved maximal effect in 40 min. Grifolic acid at the concentration of 5 μmol/L and 2.5 μmol/L also attenuated MMP in a longer 60 min. Grifolic acid at the concentration of 1.25 μmol/L did not attenuate MMP in 60 min compared to control (Fig. 3).

**Fig. 4:**
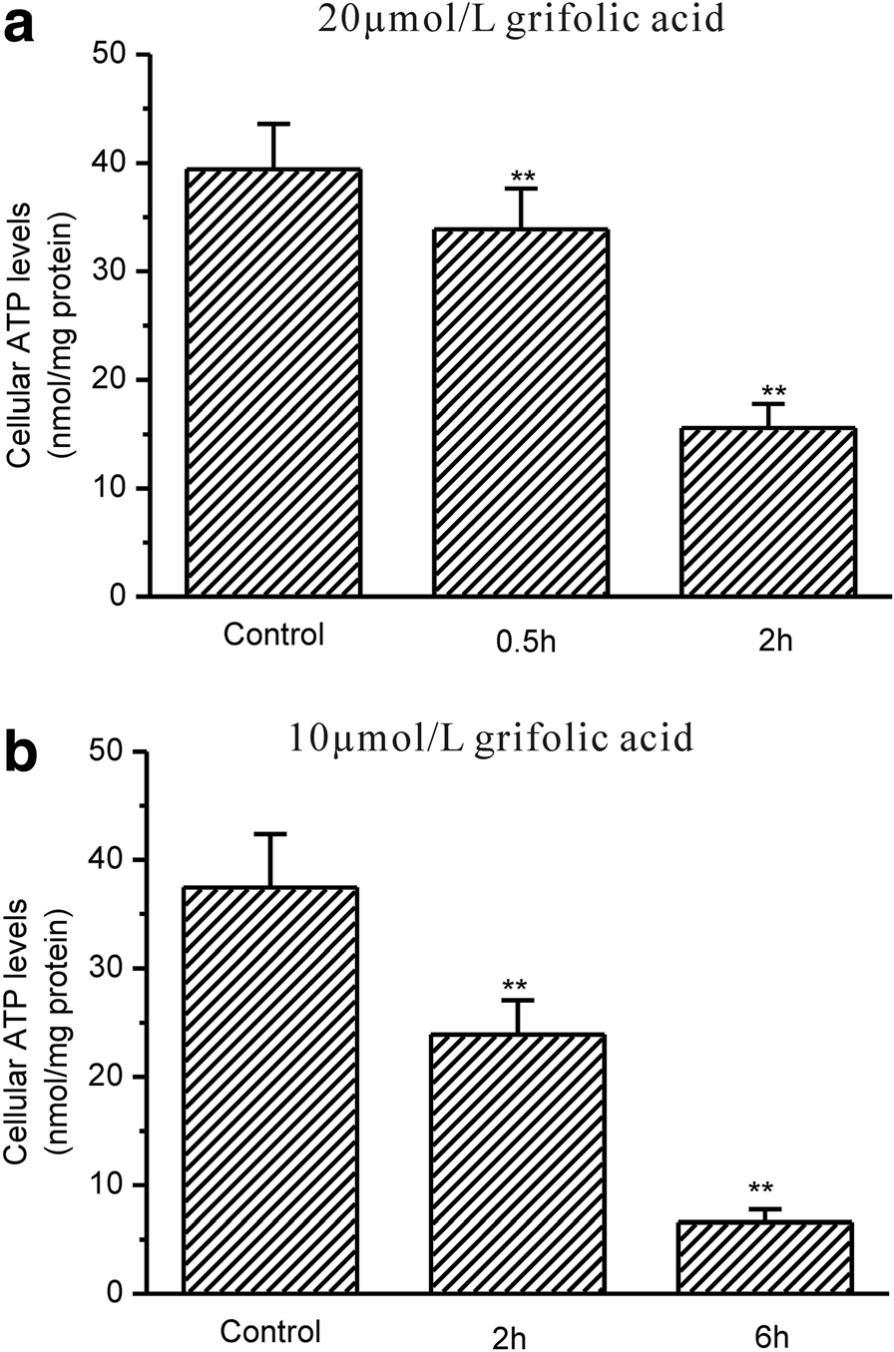
Grifolic acid reduces cellular ATP levels in GH3 cells. **a** The cellular ATP levels of GH3 cells being treated by 20 μmol/L grifolic acid; **b** The cellular ATP levels of GH3 cells being treated by 10 μmol/L grifolic acid. ** means P < 0.01 vs control, *n* = 12

The cellular ATP levels were then measured. The cellular ATP level was 38.18 ± 4.23 nmol/mg protein in control GH3 cells, and it began to significantly drop after the treatment with 20 μmol/L grifolic acid for 0.5 h and dropped to 17.76 ± 3.23 nmol/mg protein in 2 h (Fig. 4a). Grifolic acid at concentration of 10μmol/L also significantly resulted in the decrease in cellular ATP levels from 37.67 ± 4.89 nmol/mg protein in control to 22.56 ± 2.49 nmol/mg protein after 2 h of the treatment and to 8.15 ± 2.03 nmol/mg protein after 6 h (Fig. 4b).

### Role of GPR120 in grifolic acid-induced GH3 cell death

**Fig. 5:**
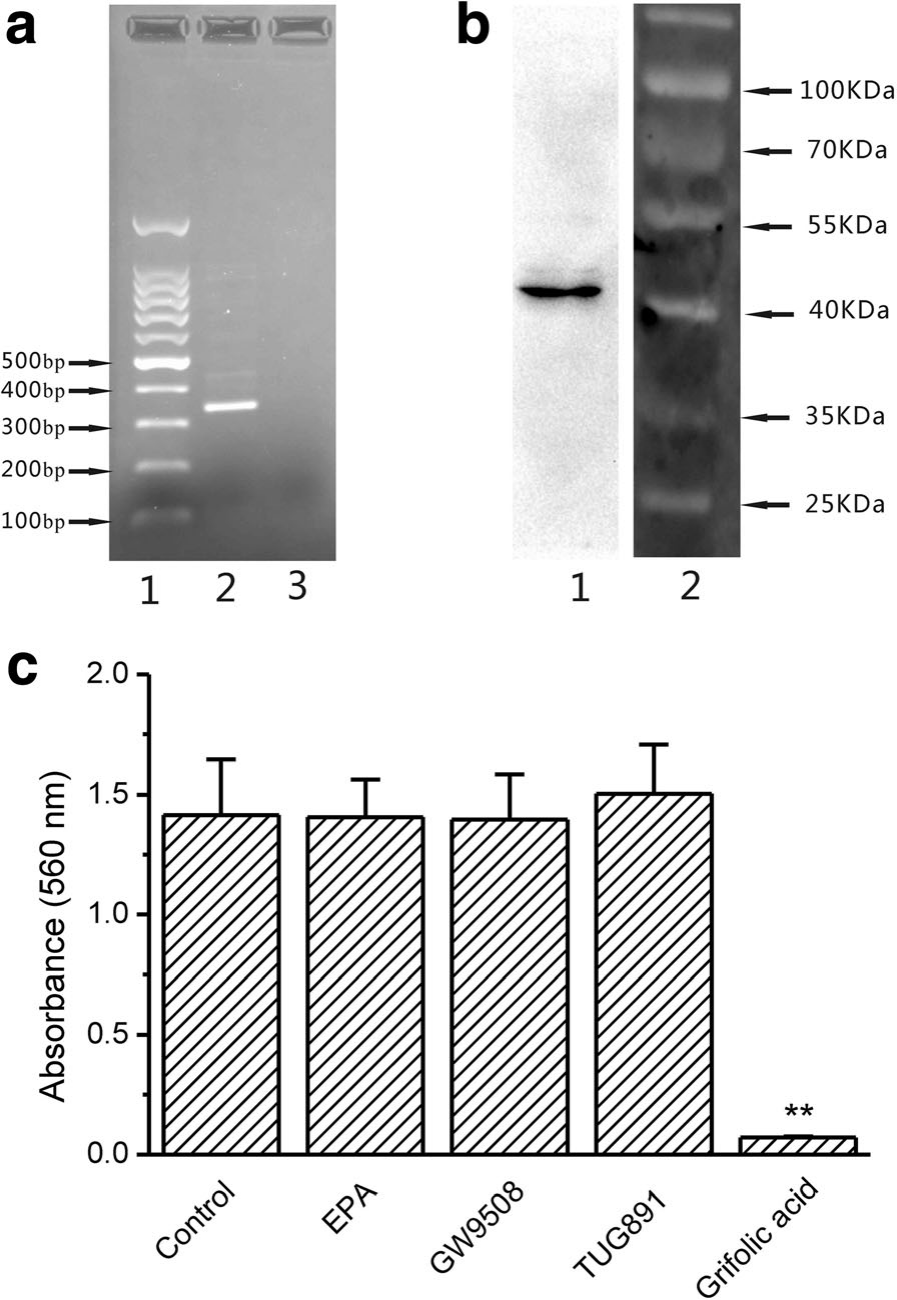
GPR120 is expressed in GH3 cells and did not influence GH3 cell viability. **a**: GPR120 transcription in GH3 cells was shown by RT-PCR. Lanes 1-3 were cDNA marker, GPR120 amplification products from GH3 cells and negative control, respectively. The size of PCR product is 340 bp. **b**: The protein expression of GPR120 in GH3 cells was shown by western blot. Lane 1 was the immunostaining of GPR120 and lane 2 was the protein marker. **c**: MTT assay of GH3 cell viability in response to GPR120 agonists and grifolic acid. ** means *P* < 0.01 vs control, *n* = 12

As shown in Fig. 5a, RT-PCR showed the expression of GPR120 in GH3 cells. The negative control using RNA without reverse transcription did not show the amplification of GPR120 gene, confirming the specificity of RT-PCR. In accordant to it, western blot showed that the proteins from GH3 cells were stained positively by GPR120 antibody and the molecular size of the stained band was 41KDa, the exact molecular size of rat GPR120 (Fig. 5b). GPR120 agonists including EPA (20 μmol/L), GW9508 (20 μmol/L) and TUG891 (20 μmol/L) did not show significant effects on the viability of GH3 cells after the incubation for 24 h. In contrast, grifolic acid (20 μmol/L) had a significant inhibitory effect on the viability of GH3 cells (Fig. 5c).

**Fig. 6:**
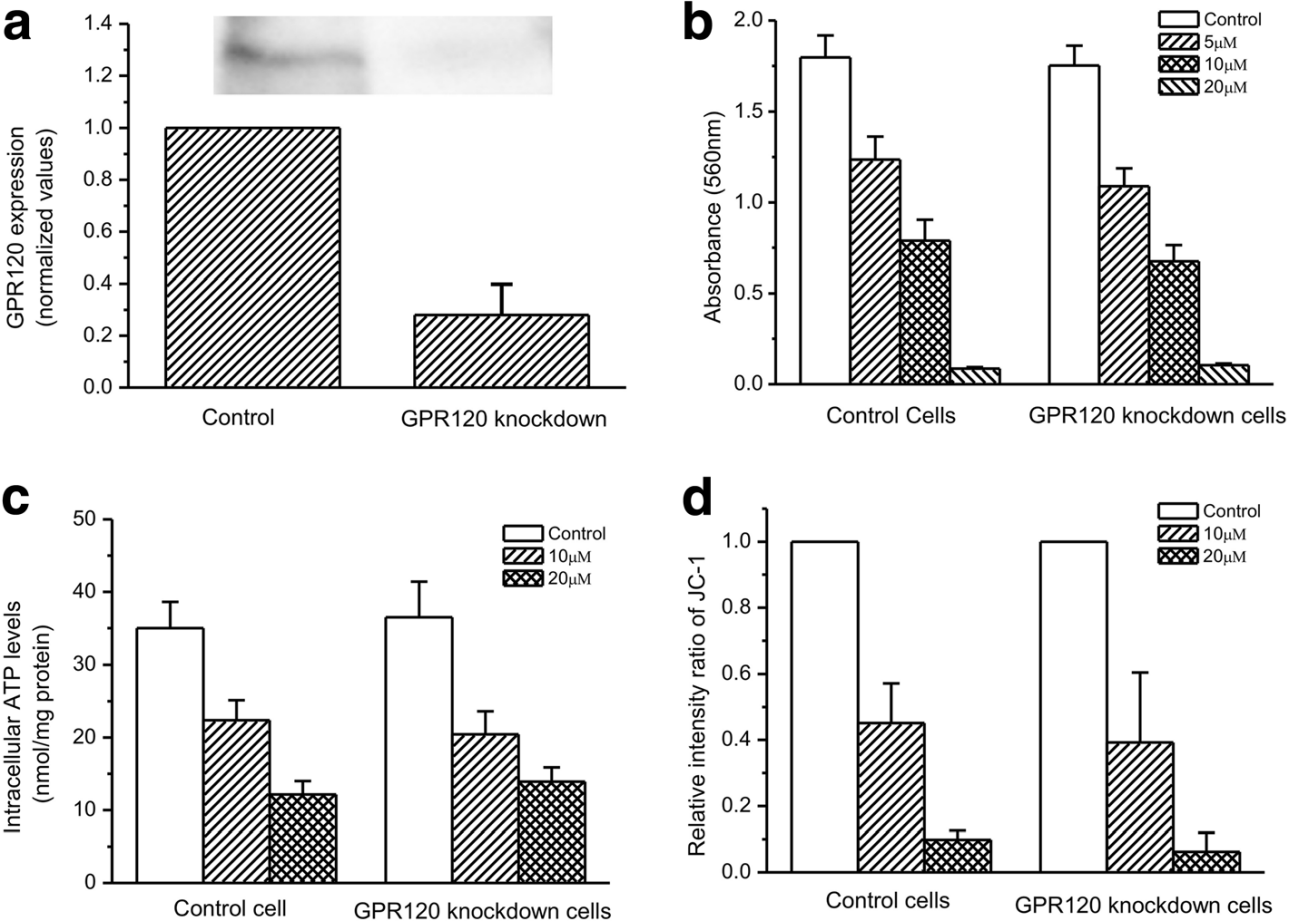
GPR120 does not mediate the effects of grifolic acid on GH3 cell viability. **a** The inhibition of GPR120 expression was achieved by siRNA transfection for 48 h; **b** Grifolic acid-induced cell death was not affected by GPR120 knockdown; **c** Grifolic acid-induced decrease in ATP production was not affected by GPR120 knockdown; **d** Grifolic acid-induced attenuation of MMP was not affected by GPR120 knockdown

A significant reduction of GPR120 protein level was achieved in GH3 cells with GPR120 siRNA transfection compared to the control (Fig. 6a). GPR120 knockdown did not significantly influence grifolic acid-induced inhibition of the GH3 cell viability (Fig. 6b). In addition, the decrease in ATP levels and MMP by grifolic acid treatment was not significantly influenced by GPR120 knockdown too (Fig. 6c and d).

### Effects of grifolic acid on electron transport chain of mitochondria and NADH production

**Fig. 7:**
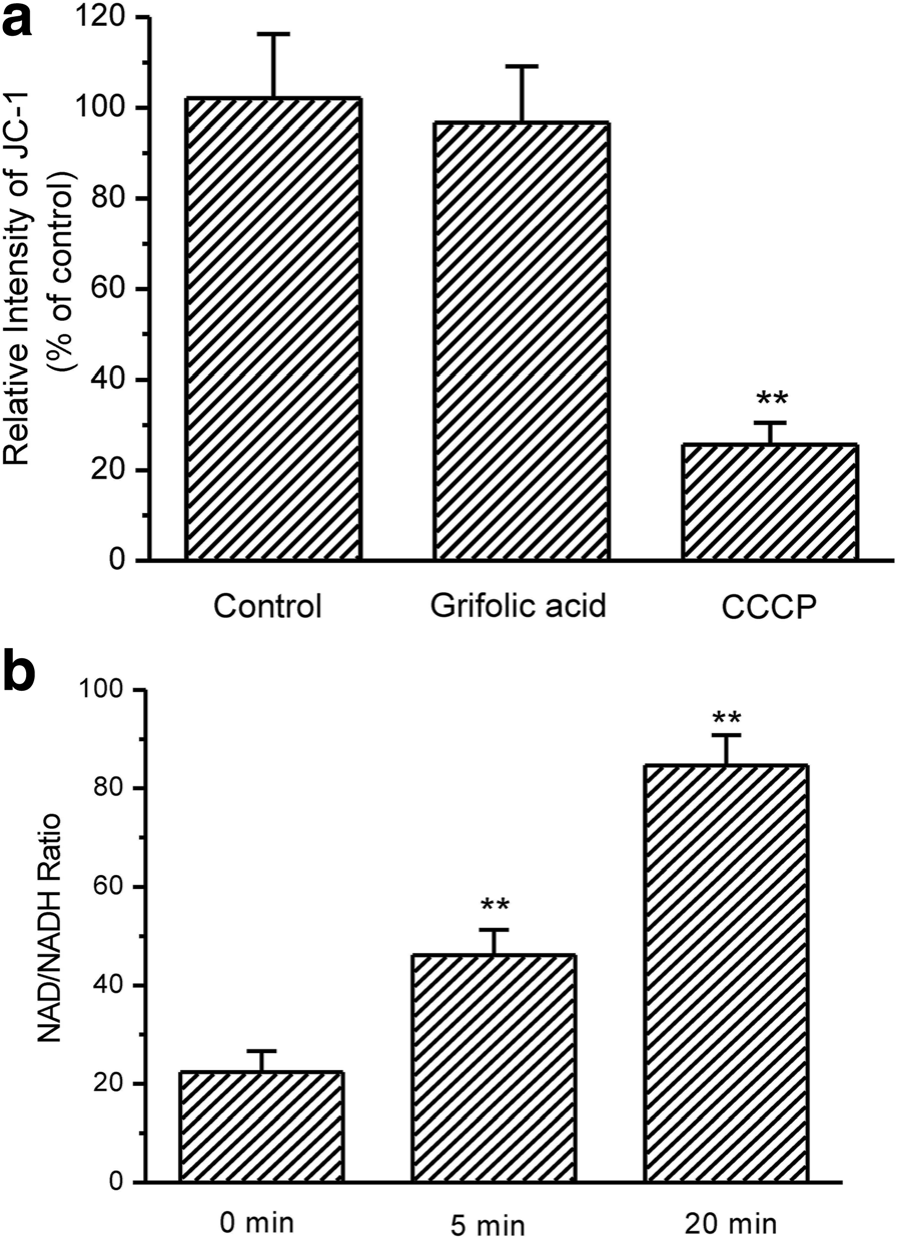
Grifolic acid does not reduce MMP of isolated mitochondria but reduces cellular NAD/NADH ratio in GH3 cells. **a** MMP of isolated mitochondria from GH3 cells was represented by JC-1 intensity. Grifolic acid (20 μmol/L) did not decrease MMP of isolated mitochondria in 20 min incubation. The uncoupler CCCP significantly inhibited MMP; **b** Grifolic acid (20 μmol/L) acutely induced a significant increase in NAD/NADH ratio in GH3 cells. ** *P* < 0.01, *n* = 6

The mitochondria from GH3 cells were stained with JC-1. JC-1 intensity of mitochondrial sample that was extracted from GH3 cells remained stable during the measurement. The uncoupler carbonyl cyanide 3-chlorophenylhydrazone (CCCP) significantly reduced JC-1 intensity of the isolated mitochondria to 25.67 ± 4.81% of the control. This result indicated that the isolated mitochondria in the experiments maintained normal MMP during the measurement. Although grifolic acid inhibited MMP in the whole cells, it did not induce any change in MMP in the isolated cell-free mitochondria during the incubation for 20 min (Fig. 7a). It is suggested that grifolic acid-induced decrease in MMP in GH3 cells is initiated by the changes prior to electron transport chain inside mitochondria.

In the next, NAD/NADH ratio in GH3 cells was measured. The NAD/NADH ratio was significantly increased after 5 min of the grifolic acid treatment and reached a higher level after 20 min, indicating that the reduction of NADH may be the reason for the decreased MMP in GH3 cells after grifolic acid treatment (Fig. 7b).

## Discussion

Grifolic acid is a farnesyl phenolic compound first isolated from the mushroom *Albatrellus confluens* and can be totally synthesized now [2]. It has been shown that grifolin, an analog of grifolic acid, has inhibitory effects on variety of tumor cells [4, 22–24]. In this study, we demonstrated that grifolic acid induced the death of GH3 pituitary adenoma cells in a dose- and time-dependent manner. Grifolic acid in concentration of 20 μmol/L induced total death of GH3 cells after 6 h treatment. In contrast, grifolin in concentration of 50 μmol/L induced only 15% of cell death after the incubation for 6 h on U2OS and MG63 osteosarcoma cells [3]. The discrepancy may result from the difference in the cell types and the culture conditions. In this study, GH3 cells were treated with grifolic acid in the serum-free medium to exclude the influence of serum ingredients. Serum-free medium may increase the sensitivity of GH3 cells to grifolic acid treatment.

It was indicated that PI3K/Akt, ERK1/2 and p53 were the possible intracellular signaling molecules that mediated the antitumor effects of grifolin in different types of tumor cells [3, 22, 24]. Although PI3K, ERK1/2 and p53 were involved in the regulation of cell viability and growth in many tumor cell types, the inhibition of PI3K, ERK1/2 and p53 did not acutely induce cell death [11, 25–28]. We also found that PI3K inhibitor Wortmannin, ERK1/2 inhibitor U-0126, and p53 inactivator cyclic pifithrin-α, p-nitro, respectively did not induce cell death in GH3 cells (Additional file 2). Therefore, it is suggested that grifolic acid-induced GH3 cell death may not be mediated by PI3K, ERK1/2 and p53.

The cellular ATP level is a critical factor maintaining cellular viability. The reduction rate of ATP production determines the cell death types [13, 29, 30]. Rapid falling of intracellular ATP leads to cell death through necrosis pathways such as oncosis [31]. Grifolic acid at 10 μmol/L induced a significant increase in the number of Annexin V and PI -positive cells, indicating that GH3 cells died mainly through necrosis. Grifolic acid also resulted in a significant and fast decrease in the cellular ATP levels in GH3 cells. The rapid deprivation of ATP may be responsible to the necrosis of GH3 cells.

Mitochondria are vital to cellular viability, with its dominant role in the production of ATP as cell energy source [32, 33]. The fuel energy is mainly transported to NADH during oxidation, and the redox energy from NADH is transferred to oxygen via the electron transport chain in mitochondria. This procedure generates MMP, which drives the protons into mitochondrial matrix through ATP synthase to produce ATP or through uncoupling proteins to produce energy. Mitochondria dysfunction may be the reason for grifolic acid-induced decrease in cellular ATP levels. We then investigated the effects of grifolic acid on mitochondrial function. MMP is usually measured using JC-1 because it accumulates in mitochondria driven by MMP to emit red fluorescence in the mitochondria and emits green fluorescence when it releases into the cytoplasm under the condition of MMP reduction [34, 35]. Grifolic acid induced a fast decrease in the red/green intensity ratio of JC-1 in GH3 cells, indicating MMP reduction or less JC-1 kept within mitochondria. Accordingly, the ATP levels in GH3 cells significantly decreased after the grifolic acid treatment. The direct action of grifolic acid on mitochondria was then studied. In the isolated mitochondria, the uncoupler CCCP, which leads to proton leak and MMP reduction [36, 37], significantly diminished MMP as expected, indicating that the isolated mitochondria may function well. Grifolic acid, however, did not reduce MMP in the isolated mitochondria of GH3 cells. It indicated that grifolic acid did not act directly on the electron transport chain of mitochondria. The MMP reduction observed in the cells may be due to the deficiency of NADH and consequent rundown of proton pump. As expected, it was found that the cellular NADH levels decreased and NAD/NADH ratio increased acutely after the grifolic acid treatment. Therefore, it is concluded that grifolic acid blocks fuel metabolism and NADH production, which in turn decreases MMP and ATP production and leads to GH3 cell death. Because fuel metabolism is executed by a series of complex enzyme reactions, the accurate targets of grifolic acid need to be further clarified in detail.

It was reported that grifolic acid is an agonist of GPR120 [7, 19, 38]. GPR120 is considered to be a promising pharmaceutical target for the treatment of metabolic diseases [39]. Recently, several non-FFA agonists of GPR120 including GW9508, TUG891, and grifolic acid have been discovered [38, 40, 41]. In this study, the mRNA and protein expression of GPR120 in GH3 cells was confirmed by RT-PCR and western blot. To our knowledge, this is the first report of GPR120 expression in GH3 cells. It was reported that GPR120 activation protects mouse enteroendocrine cell line STC-1 cells against serum deprivation-induced apoptosis [8], showing protective effects of GPR120 on cellular viability. However, we found that grifolic acid induced cell death of GH3 cells in serum-free culture condition. To clarify the role of GPR120 in the action of grifolic acid on GH3 cells, we tested the effects of the other putative GPR120 agonists on the viability of GH3 cells. Applications of EPA, GW9508 and TUG891 did not show any cytotoxic effects on GH3 cells under same conditions. In addition, GPR120 knockdown in GH3 cells did not affect the cytotoxic effects of grifolic acid. Taken together, GPR120 is not involved in the action of grifolic acid on GH3 cells. Grifolic acid was used within a range of 10-100 μmol/L in other studies to activate GPR120 [7, 19, 42, 43]. Considering that the concentration of grifolic acid used in this study is within the same range, it is more likely that grifolic acid is not a pure GPR120 agonist and have other acting targets.

## Conclusions

Grifolic acid induces GH3 cell death by inhibiting ATP production. Inhibition of mitochondrial fuel metabolism and NADH production may be the reason for the inhibition of ATP production. GPR120 is not the target of grifolic acid in GH3 cells to induce cell death and the exact signaling molecules for grifolic acid to inhibit cell viability remain to be further elucidated.

## Additional files

Additional file 1: The structure of grifolic acid. (JPG 153 kb)

Additional file 2: The effects of PI3K inhibitor, ERK1/2 inhibitor and p53 inactivator on GH3 cells. PI3K inhibitor Wortmannin (0.1μmol/L), ERK1/2 inhibitor U-0126 (1 μmol/L), and p53 inactivator cyclic pifithrin-α, p-nitro (1 μmol/L) did not induce cell death in GH3 cells respectively, as measured by MTT assay. (*P* = 0.58, *n* = 12) (JPG 2759 kb)

## Abbreviations

Cyclic pifithrin-α-p-nitro: 2-(4-Nitrophenyl) imidazo[2,1-b]-5,6,7,8- tetrahydrobenzothiazole)ATCC American Type Culture Collection
ATP: Adenosine triphosphate
CCCP: Carbonyl cyanide 3-chlorophenylhydrazone
ELISA: Enzyme linked immunosorbent assay
EPA: Eicosapentaenoic acid
ERK1/2: Extracellular regulated protein kinases
FBS: Fetal bovine serum
GIP: Glucose-dependent insulinotropic polypeptide
GPR120: G protein-coupled receptor 120
JC-1: 5,5′,6,6′-tetrachloro-1,1′,3,3′-tetraethylbenzimi- dazolylcarbocyanine iodide
MMP: Mitochondrial membrane potential
MTT: 3-(4,5-dimethyl-2-thiazolyl)-2,5-diphenyl-2-H-tetrazolium bromide
NADH: Nicotinamide adenine dinucleotide
PBS: Phosphate buffered solution
TUG891: 3-(4-((4-fluoro-49-methyl-[1,19-biphenyl]-2-yl)methoxy)phenyl) propanoic acid

## References

[1] Gerhauser C (2013). Cancer chemoprevention and nutriepigenetics: state of the art and future challenges. Top Curr Chem.

[2] Grabovyi GA, Mohr JT (2016). Total synthesis of Grifolin, Grifolic acid, LL-Z1272alpha, LL-Z1272beta, and Ilicicolinic acid a. Org Lett.

[3] Jin S, Pang RP, Shen JN, Huang G, Wang J, Zhou JG (2007). Grifolin induces apoptosis via inhibition of PI3K/AKT signalling pathway in human osteosarcoma cells. Apoptosis.

[4] Wu Z, Li Y. Grifolin exhibits anti-cancer activity by inhibiting the development and invasion of gastric tumor cells. Oncotarget. 2017;8:21454–60. 10.18632/oncotarget.15250 PMC5400597 28206955

[5] Luo XJ, Li LL, Deng QP, Yu XF, Yang LF, Luo FJ, Xiao LB, Chen XY, Ye M, Liu JK, Cao Y (2011). Grifolin, a potent antitumour natural product upregulates death-associated protein kinase 1 DAPK1 via p53 in nasopharyngeal carcinoma cells. Eur J Cancer.

[6] Hara T, Hirasawa A, Sun Q, Sadakane K, Itsubo C, Iga T, Adachi T, Koshimizu TA, Hashimoto T, Asakawa Y, Tsujimoto G (2009). Novel selective ligands for free fatty acid receptors GPR120 and GPR40. Naunyn Schmiedeberg's Arch Pharmacol.

[7] Iwasaki K, Harada N, Sasaki K, Yamane S, Iida K, Suzuki K, Hamasaki A, Nasteska D, Shibue K, Joo E, Harada T, Hashimoto T, Asakawa Y, Hirasawa A, Inagaki N (2015). Free fatty acid receptor GPR120 is highly expressed in enteroendocrine K cells of the upper small intestine and has a critical role in GIP secretion after fat ingestion. Endocrinology.

[8] Hirasawa A, Tsumaya K, Awaji T, Katsuma S, Adachi T, Yamada M, Sugimoto Y, Miyazaki S, Tsujimoto G (2005). Free fatty acids regulate gut incretin glucagon-like peptide-1 secretion through GPR120. Nat Med.

[9] Mehta GU, Lonser RR. Management of hormone-secreting pituitary adenomas. Neuro-Oncology. 2016; 10.1093/neuonc/now130 PMC5464431 27543627

[10] Molitch ME (2017). Diagnosis and treatment of pituitary adenomas: a review. JAMA.

[11] Secondo A, De Mizio M, Zirpoli L, Santillo M, Mondola P (2008). The cu-Zn superoxide dismutase (SOD1) inhibits ERK phosphorylation by muscarinic receptor modulation in rat pituitary GH3 cells. Biochem Biophys Res Commun.

[12] Seppet E, Gruno M, Peetsalu A, Gizatullina Z, Nguyen HP, Vielhaber S, Wussling MH, Trumbeckaite S, Arandarcikaite O, Jerzembeck D, Sonnabend M, Jegorov K, Zierz S, Striggow F, Gellerich FN (2009). Mitochondria and energetic depression in cell pathophysiology. Int J Mol Sci.

[13] Eguchi Y, Shimizu S, Tsujimoto Y (1997). Intracellular ATP levels determine cell death fate by apoptosis or necrosis. Cancer Res.

[14] Gonzalez VM, Fuertes MA, Alonso C, Perez JM (2001). Is cisplatin-induced cell death always produced by apoptosis?. Mol Pharmacol.

[15] van Engeland M, Ramaekers FC, Schutte B, Reutelingsperger CP (1996). A novel assay to measure loss of plasma membrane asymmetry during apoptosis of adherent cells in culture. Cytometry.

[16] Jana S, Sinha M, Chanda D, Roy T, Banerjee K, Munshi S, Patro BS, Chakrabarti S (1812). Mitochondrial dysfunction mediated by quinone oxidation products of dopamine: implications in dopamine cytotoxicity and pathogenesis of Parkinson's disease. Biochim Biophys Acta.

[17] Yu JH, Song SJ, Kim A, Choi Y, Seok JW, Kim HJ, Lee YJ, Lee KS, Kim JW (2016). Suppression of PPARgamma-mediated monoacylglycerol O-acyltransferase 1 expression ameliorates alcoholic hepatic steatosis. Sci Rep.

[18] Zhang X, Yeung ED, Wang J, Panzhinskiy EE, Tong C, Li W, Li J (2010). Isoliquiritigenin, a natural anti-oxidant, selectively inhibits the proliferation of prostate cancer cells. Clin Exp Pharmacol Physiol.

[19] Chen W, Paradkar PN, Li L, Pierce EL, Langer NB, Takahashi-Makise N, Hyde BB, Shirihai OS, Ward DM, Kaplan J, Paw BH (2009). Abcb10 physically interacts with mitoferrin-1 (Slc25a37) to enhance its stability and function in the erythroid mitochondria. Proc Natl Acad Sci U S A.

[20] Salvioli S, Ardizzoni A, Franceschi C, Cossarizza A (1997). JC-1, but not DiOC6(3) or rhodamine 123, is a reliable fluorescent probe to assess delta psi changes in intact cells: implications for studies on mitochondrial functionality during apoptosis. FEBS Lett.

[21] Perelman A, Wachtel C, Cohen M, Haupt S, Shapiro H, Tzur A (2012). JC-1: alternative excitation wavelengths facilitate mitochondrial membrane potential cytometry. Cell Death Dis.

[22] Holliday ND, Watson SJ, Brown AJ (2011). Drug discovery opportunities and challenges at g protein coupled receptors for long chain free fatty acids. Front Endocrinol.

[23] Che X, Yan H, Sun H, Dongol S, Wang Y, Lv Q, Jiang J (2016). Grifolin induces autophagic cell death by inhibiting the Akt/mTOR/S6K pathway in human ovarian cancer cells. Oncol Rep.

[24] Anbazhagan AN, Priyamvada S, Gujral T, Bhattacharyya S, Alrefai WA, Dudeja PK, Borthakur A (2016). A novel anti-inflammatory role of GPR120 in intestinal epithelial cells. Am J Physiol Cell physiol.

[25] Bavelloni A, Faenza I, Aluigi M, Ferri A, Toker A, Maraldi NM, Marmiroli S (2000). Inhibition of phosphoinositide 3-kinase impairs pre-commitment cell cycle traverse and prevents differentiation in erythroleukaemia cells. Cell Death Differ.

[26] Chattopadhyay C, Grimm EA, Woodman SE (2014). Simultaneous inhibition of the HGF/MET and Erk1/2 pathways affect uveal melanoma cell growth and migration. PLoS One.

[27] Huang HC, Chang TM, Chang YJ, Wen HY (2013). UVB irradiation regulates ERK1/2- and p53-dependent thrombomodulin expression in human keratinocytes. PLoS One.

[28] Yea SS, So L, Mallya S, Lee J, Rajasekaran K, Malarkannan S, Fruman DA (2014). Effects of novel isoform-selective phosphoinositide 3-kinase inhibitors on natural killer cell function. PLoS One.

[29] Leist M, Single B, Castoldi AF, Kuhnle S, Nicotera P (1997). Intracellular adenosine triphosphate (ATP) concentration: a switch in the decision between apoptosis and necrosis. J Exp Med.

[30] Ferrari D, Stepczynska A, Los M, Wesselborg S, Schulze-Osthoff K (1998). Differential regulation and ATP requirement for caspase-8 and caspase-3 activation during CD95- and anticancer drug-induced apoptosis. J Exp Med.

[31] Nicotera P, Leist M, Ferrando-May E (1998). Intracellular ATP, a switch in the decision between apoptosis and necrosis. Toxicol Lett.

[32] Newmeyer DD, Ferguson-Miller S (2003). Mitochondria: releasing power for life and unleashing the machineries of death. Cell.

[33] Garcia-Souza LF, Oliveira MF (2014). Mitochondria: biological roles in platelet physiology and pathology. Int J Biochem Cell Biol.

[34] Reers M, Smith TW, Chen LB (1991). J-aggregate formation of a carbocyanine as a quantitative fluorescent indicator of membrane potential. Biochemistry.

[35] Smiley ST, Reers M, Mottola-Hartshorn C, Lin M, Chen A, Smith TW, Steele GD, Chen LB (1991). Intracellular heterogeneity in mitochondrial membrane potentials revealed by a J-aggregate-forming lipophilic cation JC-1. Proc Natl Acad Sci U S A.

[36] Sureda FX, Escubedo E, Gabriel C, Comas J, Camarasa J, Camins A (1997). Mitochondrial membrane potential measurement in rat cerebellar neurons by flow cytometry. Cytometry.

[37] Wang MX, Ren LM (2006). Growth inhibitory effect and apoptosis induced by extracellular ATP and adenosine on human gastric carcinoma cells: involvement of intracellular uptake of adenosine. Acta Pharmacol Sin.

[38] Sun Q, Hirasawa A, Hara T, Kimura I, Adachi T, Awaji T, Ishiguro M, Suzuki T, Miyata N, Tsujimoto G (2010). Structure-activity relationships of GPR120 agonists based on a docking simulation. Mol Pharmacol.

[39] Yonezawa T, Kurata R, Yoshida K, Murayama MA, Cui X, Hasegawa A (2013). Free fatty acids-sensing G protein-coupled receptors in drug targeting and therapeutics. Curr Med Chem.

[40] Briscoe CP, Peat AJ, McKeown SC, Corbett DF, Goetz AS, Littleton TR, McCoy DC, Kenakin TP, Andrews JL, Ammala C, Fornwald JA, Ignar DM, Jenkinson S (2006). Pharmacological regulation of insulin secretion in MIN6 cells through the fatty acid receptor GPR40: identification of agonist and antagonist small molecules. Br J Pharmacol.

[41] Hudson BD, Shimpukade B, Mackenzie AE, Butcher AJ, Pediani JD, Christiansen E, Heathcote H, Tobin AB, Ulven T, Milligan G (2013). The pharmacology of TUG-891, a potent and selective agonist of the free fatty acid receptor 4 (FFA4/GPR120), demonstrates both potential opportunity and possible challenges to therapeutic agonism. Mol Pharmacol.

[42] Janssen S, Laermans J, Iwakura H, Tack J, Depoortere I (2012). Sensing of fatty acids for octanoylation of ghrelin involves a gustatory G-protein. PLoS One.

[43] Murase R, Sato H, Yamamoto K, Ushida A, Nishito Y, Ikeda K, Kobayashi T, Yamamoto T, Taketomi Y, Murakami M (2016). Group X Secreted phospholipase A2 releases omega3 polyunsaturated fatty acids, suppresses colitis, and promotes sperm fertility. J Biol Chem.

