# Supplementary figures and images for: Grifolic acid induces GH3 adenoma cell death by inhibiting ATP production through a GPR120-independent mechanism
Source: BMC Pharmacol Toxicol. 2018 May 30;19:26. doi: 10.1186/s40360-018-0215-4 (PMC5975534; doi:10.1186/s40360-018-0215-4)

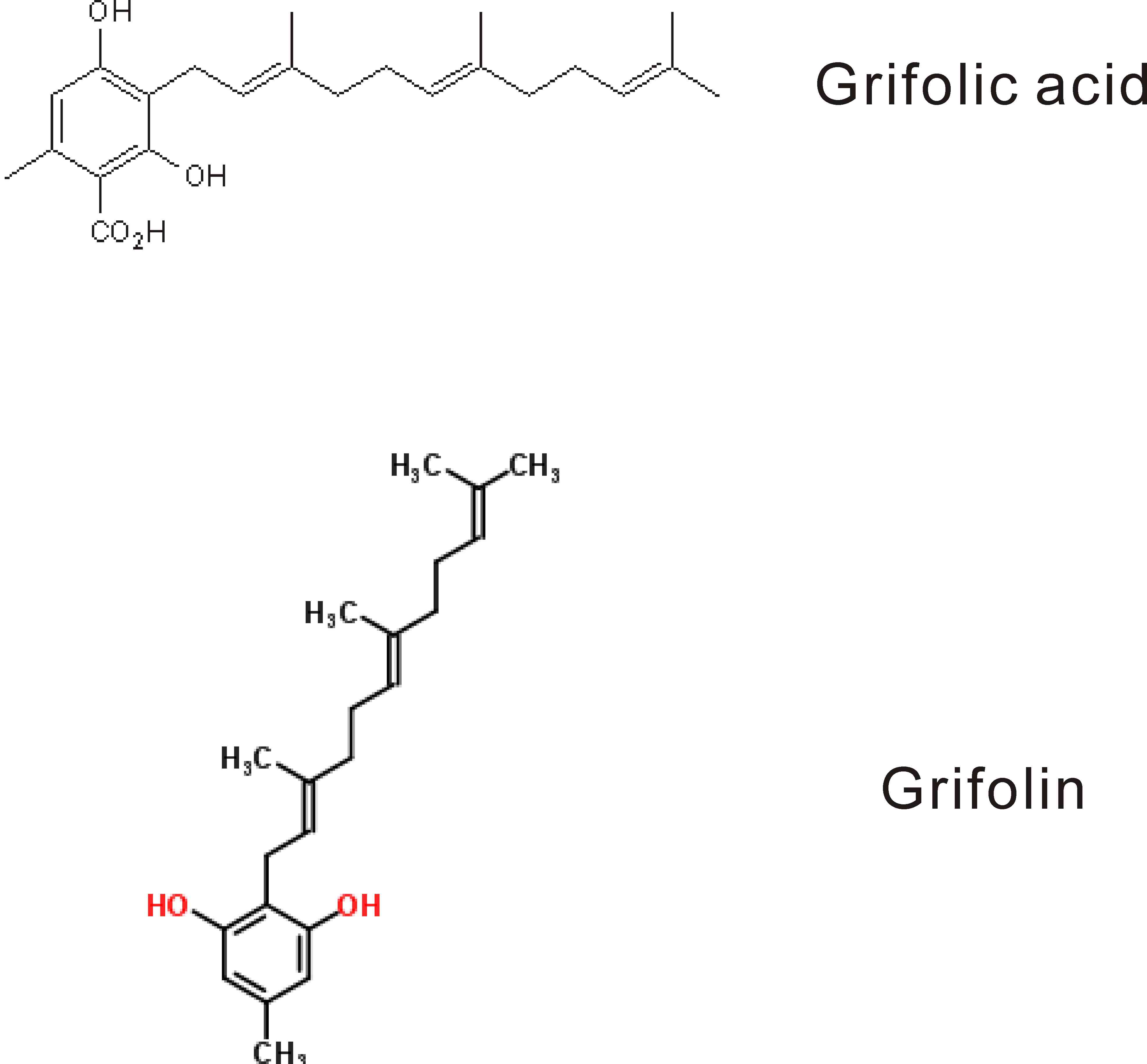

Supplement: Supplementary file 1 — The structure of grifolic acid. (JPG 153 kb) [file 40360_2018_215_MOESM1_ESM.jpg]

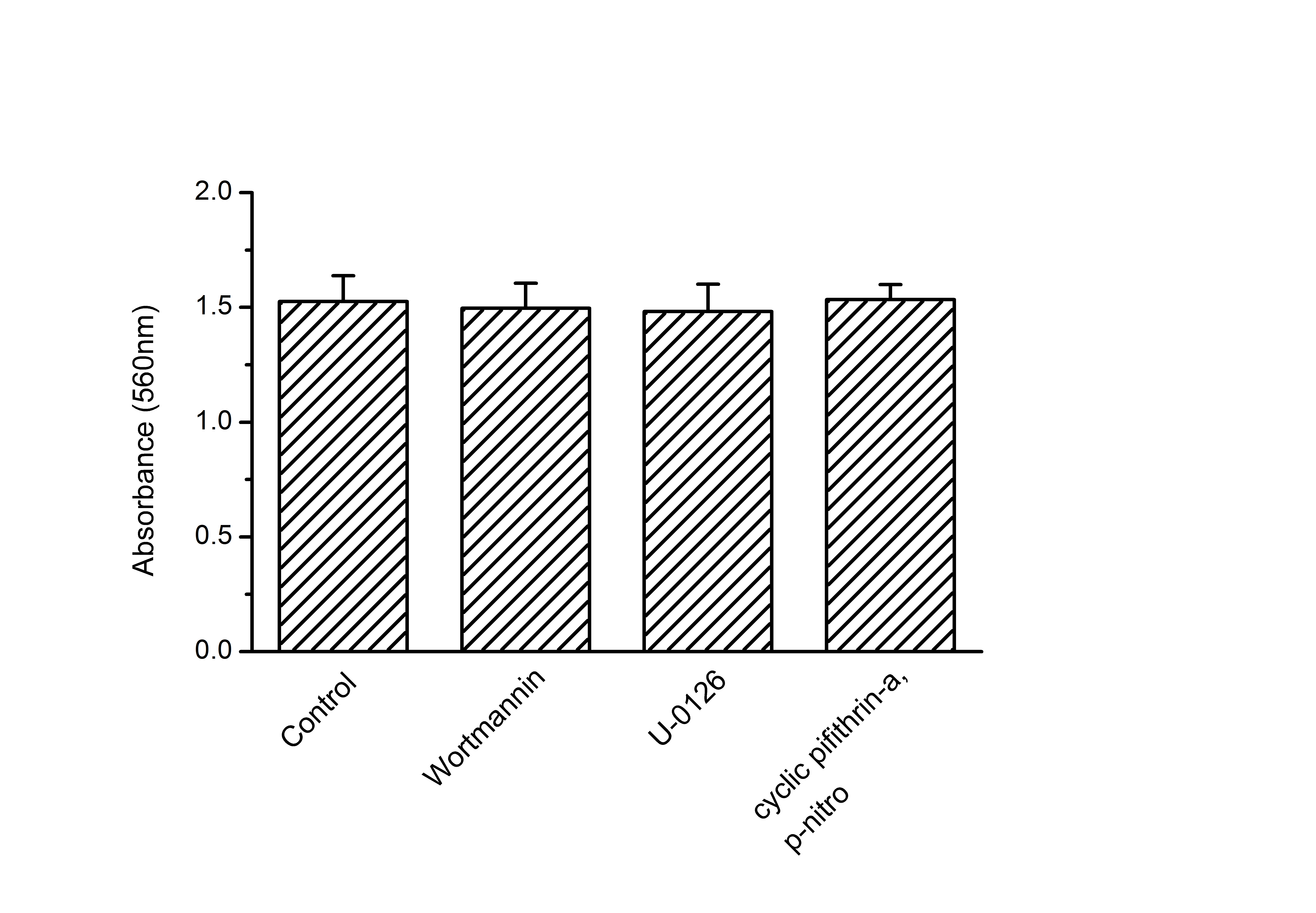

Supplement: Supplementary file 2 — The effects of PI3K inhibitor, ERK1/2 inhibitor and p53 inactivator on GH3 cells. PI3K inhibitor Wortmannin (0.1μmol/L), ERK1/2 inhibitor U-0126 (1 μmol/L), and p53 inactivator cyclic pifithrin-α, p-nitro (1 μmol/L) did not induce cell death in GH3 cells respectively, as measured by MTT assay. (P = 0.58, n = 12) (JPG 2759 kb) [file 40360_2018_215_MOESM2_ESM.jpg]
